# Community Health Risk Assessment of Total Suspended Particulates near a Cement Plant in Maros Regency, Indonesia

**DOI:** 10.5696/2156-9614-11.30.210616

**Published:** 2021-06-17

**Authors:** Annisa Utami Rauf, Anwar Mallongi, Anwar Daud, Muhammad Hatta, Wesam Al-Madhoun, Ridwan Amiruddin, Stang Abdul Rahman, Atjo Wahyu, Ratna Dwi Puji Astuti

**Affiliations:** 1 Faculty of Public Health, Hasanuddin University, Makassar, Indonesia.; 2 Faculty of Marine Science and Fisheries, Hasanuddin University, Makassar, Indonesia.; 3 Faculty of Civil Engineering and Built Environment, Universiti Tun Hussein Onn Malaysia, Malaysia.

**Keywords:** air pollution, cement plant, hazard quotient, total suspended particulates

## Abstract

**Background.:**

Cement plants generate particulate matter (PM) across processes from raw material preparation to packaging. The presence of total suspended particulates (TSP) coming out of the stack causes a high accumulation of dust in residential areas. Human exposure to TSP could affect human health and wellbeing.

**Objectives.:**

The present study aims to evaluate concentrations of TSP and to estimate the health risks of TSP exposure through the inhalation pathway in communities surrounding a private cement industry in Maros regency, Indonesia.

**Methods.:**

Total suspended particulates were collected using a high-volume air sampler (HVAS) at five locations. Samples were taken by grab sampling for 24 hours. The SCREEN3 program was used to view the maximum range and distribution of pollutants based on the geographical, stack profiles and meteorological factors in the study area. Hazard quotient (HQ) was used to estimate non-carcinogenic risks of TSP in surrounding communities.

**Results.:**

Total suspended particulate concentrations were measured with a maximum value of 133.24 μg/m^3^ and a minimum value of 18.48 μg/m^3^. This maximum value exceeds the minimum acceptable level from Canadian National Ambient Air Quality Objectives (C-NAAQOs). The non-carcinogenic risks from the inhalation pathway were low except for location 3 (HQ>1) across all locations.

**Conclusions.:**

The cement plant may significantly contribute to total TSP concentrations in air and may potentially have adverse effects on human health. Communities near the cement plant are vulnerable to TSP exposure and measures are needed to reduce TSP in Maros regency, Indonesia.

**Participant Consent.:**

Obtained

**Ethics Approval.:**

This study was approved by the Health Research Ethics Committee of Hasanuddin University with protocol number 28920093022.

**Competing Interests.:**

The authors declare no competing financial interests.

## Introduction

Air pollution has been identified as a major environmental problem associated with respiratory disease and reduced life expectancy.^1^ One indicator of reduced air quality is the presence of particulate matter (PM) which is commonly found around industrial activities, such as the cement industry.^1^,^2^ In China, the second-largest source emitter of air pollution, a cement factory contributed about 15% CO_2_ and 14% of PM <2.5 μm (PM_2.5_).^3^ In Romania, the cement industry released about 1 500 000 tons of municipal waste in 18 cities in 2004–2013, including carbon dioxide (CO_2_), hydrogen chloride (HCl) and hydrogen fluoride (HF) emission.^4^ From grinding to packaging, the entire process greatly contributes to the accumulation of total suspended particulates (TSP) inside the plant and outside the cement factory. In addition, most of the cement factory still uses coal as fuel.^5^ Emissions of total suspended particulates contain harmful pollutants, such as heavy metals, polycyclic aromatic hydrocarbons, silica, and toxic gases that easily accumulate near the cement factory.^6^,^7^,^8^

Total suspended particulate exposure is associated with respiratory infections, skin damage, and digestion problems.^9^,^10^ The accumulation of particulate matter (PM), which generally contains metals in surface plants, air, and water bodies is attributed to ecological damage.^11^ A study in China calculated exposure to PM in size <2.5 μm (PM_2.5_) and PM<10 μm (PM_10_) in terms of years of life lost (YLL) and estimated 5.2% and 6.9% of total YLL due to PM_2.5_ and PM_10_, respectively. Another study in Pakistan revealed a high rate of premature mortality associated with high concentrations of PM, with 105 000 deaths per year.^12^

The Maros-Pangkep karst ecosystem is an area with a wealth of natural resources for raw materials to supply the cement plant.^13^,^14^ The largest private cement plant in Maros regency produces 2.4 million tons of cement annually. A previous study of PM_2.5_ in the vicinity of the cement industry has been conducted but did not characterize the health risks to the community.^15^ Moreover, monitoring of water pollution in this area found that the pollution load as evidenced by total suspended solid (TSS) and chemical oxygen demand (COD) levels were extremely high.^16^ Anthropogenic activities in industrial areas have a correlation between air quality and public health status.^17^ Meteorological aspects also contribute to TSP distribution,^18^ transporting particulate matter hundreds of kilometers depending upon meteorological conditions.^19^,^20^

The study area was located near a cement plant in Baruga village, Maros regency, South Sulawesi Province, Indonesia. The cement plant is located close to residential areas (<50 m). The present study aimed to measure TSP concentrations and distribution and estimate the non-carcinogenic risk of long-term exposure of TSP to communities surrounding the cement plant. This study is expected to be a preliminary study for environmental mitigation management, monitoring and creating a plan for reducing health risks to residents in this area.

**Abbreviations**
*ADD*: Average daily dose
*HQ*: Hazard quotient
*HVAS*: High-volume air sampler
*RfC*: Reference concentration
*TSP*: Total suspended particulates
*C-NAAQO*: Canada's National Ambient Air Quality Objectives

## Methods

Maros regency is located in the western part of South Sulawesi at 5°01′04.0″ and 119°34′35.0 with an area of 1619.11 km^2^, consisting of 14 sub-districts and 103 villages. This location has sufficient rainfall, so agricultural lands are fertile. The average wind speed is 2–3 knots/hour. The highest rainfall intensity occurs in February (839 mm). The average air temperature in Maros regency is 29°C and the lowest temperature in Maros is usually recorded in May (21°C). Geographically, Maros regency is surrounded by karst which enriches this area with limestone, basalt, coal, silica sands/quartz and many other rock types.^21^ There are many mining and industrial operations in this area, such as cement industry and limestone mining.

### Sampling

An ambient air test sample was taken in November 2020. The maximum and dominant winds were blowing towards the residential areas in this month over the last 5 years (2015–2019). October–March is the rainy season in Maros and is also windy. Air sampling cannot be handled in October because of the high frequency of rainfall. Taking into consideration weather, rainfall and wind data, November is a representative time to collect the study data. This sampling time represents the rainy season for TSP exposure. Sampling was performed over 5 days from 23–27 November 2020 (weekdays), assuming the activity of cement factory is nonstop for 24 hours and dust is continually generated from the stacks. Measurements were carried out around the residential area of the private cement plant. The ambient air quality analysis used the Indonesian National Standard (SNI) 7119-3: 2017.^22^ Sampling was performed with a high-volume air sampler (HVAS) (*TFIA 2 HVAS Staplex*) placed 1.5 meters above the ground. The device was placed at each sampling site and connected to a power source. Average flow rate was set at a speed of 1.5 m^3^/minute. Collection time and coordinates were recorded. All locations are the closest residential areas to the cement factory. The distance of location 1 from the cement factory is 2520 m, location 2 is 817 m, location 3 is 642.9 m, location 4 is 2210 m and location 5 is 5317 m.

Daily meteorological supporting data were accessed at the Meteorological, Climatological and Geophysical Agency (BMKG) Online Database Center. Data included temperature, humidity, rainfall, and wind direction. Sampling was conducted in open areas within 500 m–850 m of the road to reduce bias from vehicle fumes. Ambient air dust was collected in the form of total suspended particulates (TSP) for 24 hours. Samples were stored and coated with aluminum foil before being moved to the laboratory. Samples were analyzed at Center of Plantation-Based Industry (BBIHP), in Makassar, South Sulawesi Province Indonesia. The particulates trapped in the HVAS were then weighed.

### Dispersion modeling

Dispersion models are commonly run through a computer program with user interfaces for entering and viewing data. Gaussian plume modeling is most commonly used.^23^ Based on mathematical equations and fundamental assumptions, the model is able to estimate plume behavior. In a rural environment, stack emissions to air and their impact on ambient air quality are important. The necessary variables are emission rate, stack height, stack inside diameter, gas exit temperature and ambient air temperature. The probability of pollutant dispersion from the two stacks were collected from stack profiles. A receptor is defined as any receptor located above the ground level. In the present study, receptors were humans living near the cement plant.

Wind speed and stability class affect maximum ground level concentrations. Meteorological and supporting data were obtained from the BMKG and internal data from the cement plant. Wind is a major factor in the distribution of TSP in this study, so it is important to visualize its direction. A wind rose is a graphical tool used by meteorologists to provide an overview of wind speeds and direction in a particular location. Color bands indicate wind speed ranges. The longest spoke shows the wind direction with the greatest frequency. The wind rose plot was processed with Microsoft Excel software and WRPLOT 4.0.1. For TSP concentration, data were visualized using ArcGIS 10.8. To estimate the distance over pollutant concentration, the SCREEN3 air dispersion model was used.

### Human data sampling

Human data were obtained through individual interviews by going door to door at respondents' houses. The research implementation permit was obtained from local authorities and the Health Research Ethics Committee of Hasanuddin University with protocol number 28920093022. Anthropometric measurement data (weight and height) were taken from 250 respondents. Human sampling was carried out by random cluster sampling. All respondents were residents who had been living, working or studying in the study area for at least a year as several factories and schools are located in the vicinity of the sampling area. All participants provided written informed consent prior to enrollment in the study.

### Health risk assessment

Health risk assessment of TSP exposure through the inhalation route was performed among communities in residential areas from <50 m – 5 000 m near the private cement plant. Five locations were chosen according to residential location and wind direction. The United States Environmental Protection Agency (USEPA) method was applied to obtain average daily dose (ADD) and hazard quotient (HQ) in Equations 1 and 2.


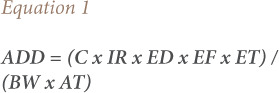


Where C is the TSP concentration (μg/m^3^); IR is the inhalation rate (adult: 0.83 m^3^/hour);^24^ ED is the exposure duration (years); ET is exposure time (hours/day); EF is exposure frequency (day/year); BW is body weight (kg); and AT is average time (ED x 365 days for non-carcinogenic effect). In the present study, ED is the amount of time for daily exposure (24 hours/day for residential exposures),^24^,^25^ EF is the frequency of annual exposure (residential exposure: 360 days/year),^25^ and ET refers to the length of time the cement plant has been in operation (20 years). The C value is the concentration of TSP in the present study and BW is body weight (kg) of each resident from anthropometric measurements collected by interview. Determination of non-carcinogenic risk was performed by calculating the HQ value.^17^ The ADD value is divided by the reference concentration (RfC). The RfC is the reference concentration for the inhalation route (mg/m^3^).^25^ The RfC value in this study is 20 μg/kg/day.^24^ An RfC expressed in μg/kg/day would be equal to the average daily dose (μg/kg/day).


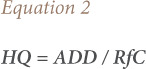


An HQ value >1 indicates no non-carcinogenic risk to the population with negligible risks to human health. Body weight data was used to calculate the intake (ADD) for individual health risks. Two hundred and fifty (250) subjects agreed to participate in the present study. Table 1 shows the values of several variables required for determining the population health risk based on the environmental health risk assessment method.^26^

**Table 1:** Human Health Risk Assessment Variables

**Parameters**	**Values**				

	Location 1	Location 2	Location 3	Location 4	Location 5
Mean of body weight (kg)	55.84	54.90	56.70	54.39	57.44
Mean of TSP concentration (μg/m^3^)	42.90	37.74	133.24	31.77	18.48
IR (m^3^/hour)			0.83^24^		
ED (years)			24^26^		
ET (hours/day)			20^26^		
EF (day/year)			360^26^		
AT (days)	30 × 365 = 10965 days (30 years for non-carcinogenic risk)				

Abbreviations: AT, average time; C, TSP concentration; ED, exposure duration; EF, frequency of annual exposure; ET, length of time cement plant has been in operation (20 years); IR, inhalation rate.

## Results

Meteorological data, including air temperature, humidity, rainfall, wind direction, and wind speed are shown in Table 2. Concentration and meteorological data were obtained at five locations. Site selection was made according to ground level of emissions and wind direction. The highest concentration was recorded at Location 3 and the lowest at Location 5.

**Table 2:** Total Suspended Particulate Concentration, Temperature, Humidity, Rainfall and Wind Speed

**Location**	**TSP concentration in 24 hours (μg/m^3^)**	**Temperature (°C)**	**Humidity (%)**	**Rainfall (mm)**	**Wind Speed (m/s)**
1	42.90	28.40	86	1.20	2.20
2	37.74	27.20	88	1.00	2.20
3	133.24	26.60	88	1.20	2.00
4	31.77	27.10	90	1.40	2.40
5	18.48	28.20	85	0.80	2.80

*Mean*	52.82	27.50	87.4	1.12	2.32

*Min*	18.48	26.60	85	0.80	2.00

*Max*	133.24	28.40	91	1.40	2.40

**Figure 1:**
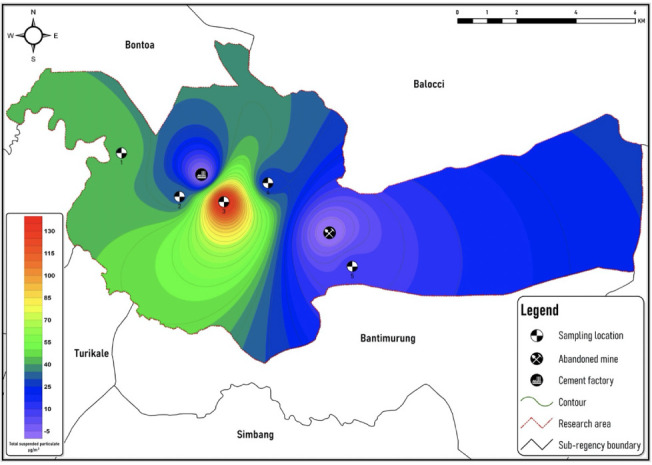
Concentration of total suspended particles in study area

The distribution of TSP concentrations in the study location are shown in Figure 1. The highest concentration of TSP was recorded in Tukamasea village, located southeast of the cement plant. Furthermore, the lowest concentration of TSP was recorded in Leang-Leang village.

### Dispersion modeling

**Figure 2:**
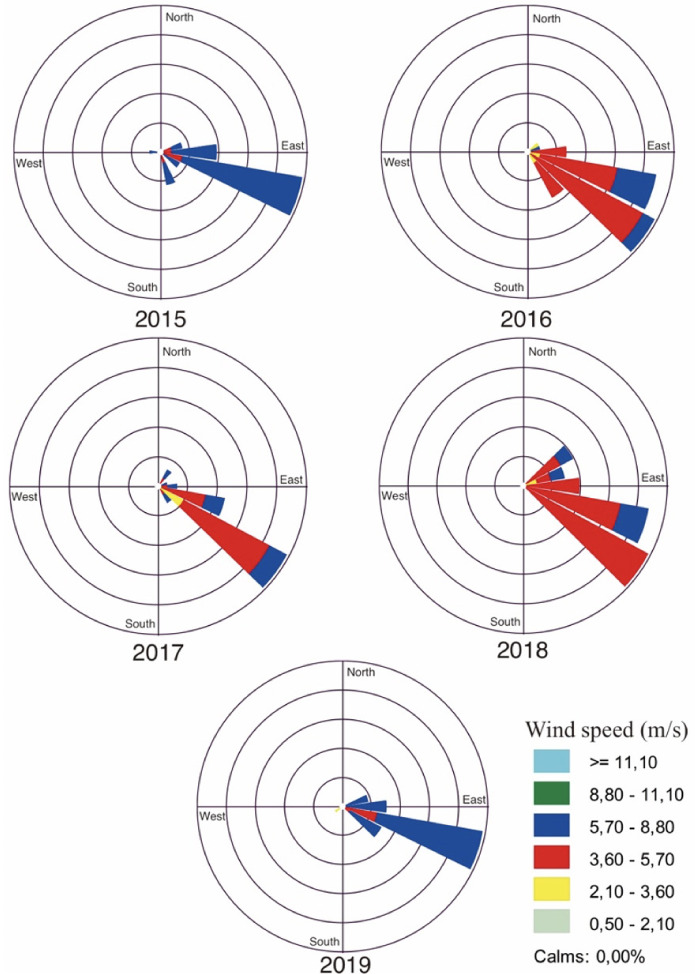
Rose diagrams based on wind frequencies in November (2015–2019)

Figure 2 is a wind rose visualization created by dominant wind direction using data from November data over a period of five years (2015–2019) in Maros regency. The resultant vector was out the southwest at 106-117°. Although the wind direction might change from day to day, generally, wind patterns were fairly stable from 2015–2019. The locations chosen were Salenrang village, Baruga village, Tukamasea village, Bungaeja village and Leang-Leang village. These are residential areas around the cement operation.

The cement plant has two stacks using coal to operate, Raw mill 1 and Raw mill 2. Raw mills are the place where crushed raw materials are mixed and stored for homogenization.^27^ We used data from raw mills to measure emission sources. As the point of emission sources, important information such as coordinates to locate its position, height from the ground, released gas, emission rate, internal stack diameter and output temperature are presented in Table 3.

**Table 3:** Cement Factory Stack Profiles

**Variables**	**Raw Mill 1**	**Raw Mill 2**
Coordinates	S: 04°56′37.4″	S: 04°56′41.75″
	E: 119°37′37.1″	E: 119°37′32.01″
Elevation	19m	24 m
Stack height	60 m	155 m
Temperature	130.30°C	81°C
Gas exit velocity	7.53 m^3^/s	6.49 m^3^/s
Diameter	5.74 m	4.80 m
Debit	194.93 m^3^/s	117.58 m^3^/s
Total Suspended	56.64 mg/Nm^3^	31.93 mg/Nm^3^
Particulates (TSP)		
Emission rate	0.43 g/s	0.21 g/s

Abbreviation: TSP, total suspended particulates

**Figure 3:**
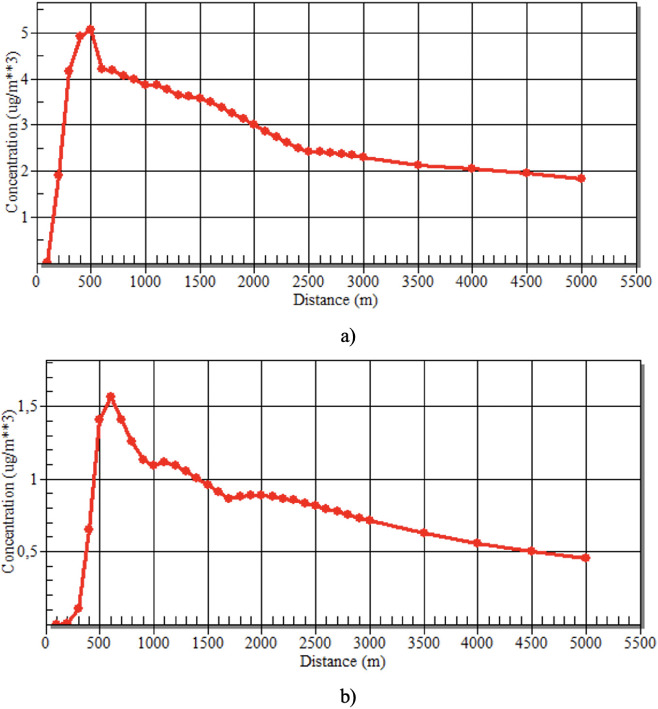
Automated distance and concentration levels in raw mill 1 (a) and raw mill 2 (b)

Figure 3 shows the results of the data interpretation of the air dispersion model obtained from variables in Table 2. The results indicated that the farther the particulate distribution, the lower the particulate concentrations. The range of 500–600 m showed the highest particulate accumulation from both stacks. Dispersion model prediction is sensitive to changes in height and wind shear.^23^

### Health risk assessment

**Figure 4:**
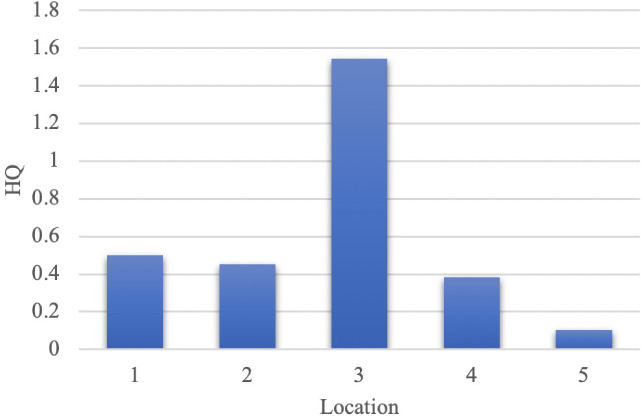
Hazard quotient

The risk level is expressed in terms of the HQ. Previously, it was necessary to calculate the intake via the inhalation pathway (ADD). Intake is the inhaled concentration of pollutants per kilogram of body weight^28^ and RfC is baseline data for no health effects due to lifetime exposure. Figure 4 shows that the highest risk was in Location 3 (HQ>1), in Tukamasea village. Meanwhile, in Locations 1, 2, 4 and 5, no health adverse effects were indicated (HQ<1). High concentrations of TSP were found at Location 3 due to the fact that this area is the closest to the wind blowing to the southeast.

## Discussion

In Table 4, the results of the present study are compared to existing standards and previous studies. Total suspended particulate measurements in all locations showed that Location 3 (max) had the highest level of TSP which can not to be tolerated according to maximum daily exposure limits, while other locations were below the threshold limit of the Canadian National Ambient Air Quality Objectives (C-NAAQOs).^29^ A previous study found that heavy metals were present in the soil surface close to the cement plant.^14^,^30^,^31^ The concentration of TSP in Maros was slightly similar to the concentration of TSP in the nearest area around the Khouzestan cement company in Iran,^32^ and less than the average TSP concentration in Mexico.^31^ A previous study around a cement plant in Jordan also found 142,8 μg/m^3^ of TSP concentration over 24 hours.^33^ Higher particulate matter concentrations may indicate increased concentrations of bounded heavy metals. In a study in China, TSP contained lead (Pb), cadmium (Cd), chromium (Cr), manganese (Mn), zinc (Zn), arsenic (Ar), nickel (Ni) and copper (Cu).^34^

**Table 4:** Comparison of Total Suspended Particle Concentrations to Standards and Previous Studies

	**Concentration (present study)**	**WHO 24 hours^36^**	**C-NAAQO 24 hours^29^**	**Jordanian Standard 24 hours^37^**	**González et al^31^**	**Sobhanardakani and Suedi^32^**
**TSP**	mean: 52.82 μg/m^3^	PM_2.5_: 25 μg/m^3^	120 μg/m^3^	260 μg/m^3^	84,73 ± 12,85 μg/m^3^	137.17±16.45 μg/m^3^
	min: 18.48 μg/m^3^	μg/m^3^				
	max: 133.24 μg/m^3^	PM_10_: 50 μg/m^3^				

Abbreviations: TSP, total suspended particulates, WHO, World Health Organization, Canadian National Ambient Air Quality Objectives, C-NAAQOs

Diseases occurring from human particulate matter exposure are asthma, bronchiolitis and chronic obstructive pulmonary disease.^35^

### Dispersion modeling

Figure 2 shows the wind direction distribution. Based on wind distribution data in November for five years (2015–2020), the average wind direction is to the east and southeast with an average speed of 2.56 m/s. Due to weather conditions, dominant winds in this study area were heading to the east, east-southeast, and southeast directions. Location 3 and 4 are categorized as locations with high wind frequencies to the east and east-southeast. The winds around Location 1 and 2 followed the direction of the west wind. The western part of this area was less affected by wind distribution. On the other hand, Location 5 is away from the main emission source. Wind direction greatly affects transport and dispersion processes.^20^,^38^ Apart from meteorological and anthropogenic factors, other factors, such as natural vegetation, have a strong influence.^35^ Total suspended particle levels are possibly higher when the wind is blowing to the southeast compared to wind from other directions.

Areas where wind is persistent will possibly experience higher pollutant concentrations even though other areas are within the same distance from the pollutant source.^19^,^39^ Tukamasea and Bungaeja village are located in the prevailing southeast wind direction. If the wind speed is stronger, the distribution will reach further, with decreasing concentration.^19^,^23^ Pollutant concentrations were positively correlated with humidity.^40^ Wind distribution can cause particulates to move and spread over the area and accumulate over a long duration.^40^ Seasonal change is the main factor in TSP distribution, as the rainy season is characterized by lower dust concentrations than the dry season. In addition, the rainy season is significantly associated with other factors, such as humidity and temperature. The high kinetic energy of raindrops is able to remove a significant amount of particulate matter.^41^ In Johannesburg, winds blowing from the source of the pollutant to surrounding communities increased the likelihood of higher dust exposure compared to winds blowing in the opposite direction.^42^ The wind conditions which blow towards the settlement can be combined with the pollutant dispersion model to estimate the range of pollutants in the study area using a mathematical model simulation. By entering the specific characteristics of the study area such as local topography, fumigation and receptor height, accurate results can be obtained.

The Gaussian air pollution model from the SCREEN3 program was used to view the predicted concentration of air pollutants and the possible range of dust distribution. The maximum pollutant concentration was in the peak range about 500–700 m from the stacks (*Figure 3*). The present study chose a range between 1 m–5000 m from the stack to determine the effect of distance on TSP accumulation. Areas that are more distant from pollutant sources (>1500 m) will be safer to live in. This is consistent with the findings from similar studies in the vicinity of other cement factories. In a study around the cement industry in Amman, Jordan, it was found that TSP concentrations at less than 500 m exceeded the Jordanian Standard (JS 1140/2006).^37^ Another study by Gholampour reported that wind direction and season played an important role. In winter, the concentration of particulate matter will be higher due to the influence of thermal inversion, decrease in temperature, or increase in the frequency of calm wind.^20^ The maximum radius of air pollutant dispersion around the cement industry using AERMOD is up to 3 kilometers.^43^

Modeling results can be used for planning new facilities in an appropriate area.^23^ Companies can adjust initial stack height, coal use and monitor the surrounding environment. In the present study, the most likely action involves designing mitigation strategies and evaluating existing policies. Maros-Pangkep is a karst area with abundant sources of limestone, basalt and alluvial deposits.^14^ Associated industrial activities can lead to production of byproducts that are harmful to the environment. In this situation, an advanced deployment model may be more suitable for the situation and get better results. Long-term exposure to TSP is harmful to human health, especially dust directly inhaled by local residents next to the industrial area.^20^

### Health risk assessment from nearby communities

In determining the level of health risk, a higher hazard quotient value indicates health risks to the surrounding population. In Figure 4, the highest HQ was in Location 3 (HQ 1.54), followed by Location 1 (HQ 0.50), Location 2 (HQ 0.45), location 4 (HQ 0.38), and Location 5 (HQ 0.10). Total suspended particulates, both PM_2.5_ and PM_10_, are able to penetrate the respiratory system.^35^ Frequent exposure can cause respiratory problems, decreased lung capacity, cardiovascular disease and mortality.^35^,^44^ Some studies have shown a relationship between TSP and adverse health effects.^32^ Location 5 was an area with the lowest hazard quotient value. This may be influenced by distance from the emission source and having the lowest TSP concentration. In this area, vegetation might play an important role, even though activities of the population may be similar to other locations.

Total suspended particulates often accumulate in environmental media such as soil and water bodies.^45^ In plants, TSP will interfere in the plant photosynthesis process. The accumulation of TSP from the cement industry is dangerous because it potentially contains harmful particulates that are carcinogenic elements.^46^,^47^ The accumulation of dust around the cement industry in Brazil was found to cause physical and chemical changes in cactus and *Cenchrus ciliaris L*. They found chlorotic spot, shorter stem, cells thickening, and leaf curling.^48^,^49^ The cement crust, becoming a phytotoxic pollutant, blocked 30–50% light onto *C. fissilis*.^50^ In this research, the farther the particulate distribution, the lower the amount of particulates in the air. Furthermore, supporting meteorological factors, such as wind direction played an important role.^51^,^52^

A study on the health impact of TSP exposure near the cement industry in Zambia, the incident rates of reported respiratory symptoms were higher than the control (uncontaminated area) and able to decrease lung function.^53^ In early childhood, exposure to TSP increases the risk of pneumonia, especially in PM_2.5_ form and its constituents.^54^ A study found a non-carcinogenic risk of dust exposure through hand-mouth intake to children in industrial areas in north China.^9^ If non-carcinogenic and carcinogenic risks occur from an early age, higher frequency and longer exposures will have more harmful effects. The values for non-carcinogenic risk were obtained using dose response data from epidemiological data standards and actual intake.^25^ This research could assess pollutant exposure events and probabilistic risks that might occur even with low concentrations and intakes which potentially have consequences for public health in the future.^55^ Carcinogenic factors from particulate matter exposure through inhalation can be influenced by the content of particulates containing harmful inorganic substances.^56^,^57^ An association was found in Kuwait between particulate matter and premature adult mortality.^58^

The private cement factory in Maros regency, Indonesia potentially produces large amounts of pollutants such dust, fly ash, poisonous gas, and other hazardous substances which pose a threat to the environment. Cement companies should begin to replace coal combustion with the latest processes and cement production composition to reduce environmental pollution from particulate matter (PM), CO_2_, NOx and SO_2_.^60^,^61^,^62^ Cement industries are expected to manage existing emissions and determine appropriate stack heights.^23^ Meanwhile, local residents should reduce their outdoor activities when wind speed is high and dominant towards residential areas, especially those living in Location 3 (HQ>1). In addition, personal protection equipment such as masks should be used. Face masks have been shown to protect wearers from inhalation of fine particulate matter and viruses.^62^

### Study limitations

Calculated health risks only represent risk in the rainy season. We recommend further research on TSP exposure in the dry season to compare the risk between the rainy and dry season.

## Conclusions

The present study was carried out to determine levels of TSP and the influence of distance variations in meteorological factors for TSP concentrations near a cement plant in Maros regency, Indonesia. In sampling locations, the 24-hour concentrations of TSP exceeded the C-NAAQOs in Location 3. The present study successfully combined an examination of the potential health risk of TSP exposure and provides information on the possible range of particulate distribution through Gaussian air dispersion modeling using SCREEN3. The results show that the modeling approach is a useful tool for estimating the distance of pollutant dispersion by using specific data such as pollution source position, meteorological status and geographic condition in residential areas. Cement production activities have possibly affected ambient air quality. Increasing TSP concentrations will elevate the non-carcinogenic risk of respiratory illness and other diseases for residents near the cement plant. The longer the duration of outdoor activities, the higher the health risk of TSP exposure through inhalation. This could lead to the emergence of chronic respiratory diseases with longer duration of exposure.
